# Is So Called “Split Alpha” in EEG Spectral Analysis a Result of Methodological and Interpretation Errors?

**DOI:** 10.3389/fnins.2020.608453

**Published:** 2020-11-26

**Authors:** Ewa Zalewska

**Affiliations:** Nalecz Institute of Biocybernetics and Biomedical Engineering, Polish Academy of Sciences, Warsaw, Poland

**Keywords:** electroencephalography (EEG), spectral analysis, Fast Fourier Transform (FFT), spurious peaks, Gibbs phenomenon

## Abstract

This paper attempts to explain some methodological issues regarding EEG signal analysis which might lead to misinterpretation and therefore to unsubstantiated conclusions. The so called “*split-alpha*,” a “new phenomenon” in EEG spectral analysis described lately in few papers is such a case. We have shown that spectrum feature presented as a “*split alpha*” can be the result of applying improper means of analysis of the spectrum of the EEG signal that did not take into account the significant properties of the applied Fast Fourier Transform (FFT) method. Analysis of the shortcomings of the FFT method applied to EEG signal such as limited duration of analyzed signal, dependence of frequency resolution on time window duration, influence of window duration and shape, overlapping and spectral leakage was performed. Our analyses of EEG data as well as simulations indicate that double alpha spectra called as “*split alpha*” can appear, as spurious peaks, for short signal window when the EEG signal being studied shows multiple frequencies and frequency bands. These peaks have no relation to any frequencies of the signal and are an effect of spectrum leakage. Our paper is intended to explain the reasons underlying a spectrum pattern called as a “*split alpha*” and give some practical indications for using spectral analysis of EEG signal that might be useful for readers and allow to avoid EEG spectrum misinterpretation in further studies and publications as well as in clinical practice.

## Introduction

Recently, few papers have been published describing a “*new phenomenon*” in EEG signal spectral analysis called “*split alpha.*” What the respective authors mean by this is “*the presence of two or more peaks with close frequencies in the EEG frequency alpha band*” (Olejarczyk et al., 2017). This term has been coined by Robinson et al. (2003) in their modeling calculations using a model of corticothalamic system when incorporating parameter non-uniformities into his previously developed uniform model (Robinson et al., 2001, 2003) and developed in further studies of Robinson’s group (Robinson et al., 2001, 2003; O’Connor and Robinson, 2004; Xiong and Yao, 2005; Gray and Robinson, 2013). Chiang et al. (2008, 2011) developed a method for the automatic identification of multiple alpha peaks in EEG data.

In 2017, Olejarczyk et al. in the paper “*The EEG Split Alpha Peak: Phenomenological Origins and Methodological Aspects of Detection and Evaluation*” presented some calculations on EEG signal which were to explain these theoretical speculations of Robinson et al. (2001, 2003) on the so called “*split alpha.*” The authors have claimed to show a “*phenomenological origins*” and also “*methodological aspects of detection and evaluation.*” In order to achieve that, the authors have analyzed EEG recordings “*using spectral analysis as well as Directed Transfer Function, a method used to evaluate functional brain connectivity.*” They tried to “*test the impact of window size and choice of reference electrode on the identification of two or more peaks with close frequencies in the spectral power distribution, so called ‘split alpha.*”’

Unfortunately, authors have improperly interpreted results of calculations and have drawn unsubstantiated conclusions. Therefore, this topic requires explanation. Other methodological errors found in the cited paper including use of Directed Transfer Function (DTF) method for “*split alpha*” generators localization, are out of the scope of this paper since spectral analysis is a key problem.

In conclusions the authors stated that “*the split alpha spectrum can be generated by two or more independent and interconnected alpha wave generators located in different regions of the cerebral cortex, but not necessarily in the occipital cortex*” and “*a window size of 2 s was found to be optimal for this purpose*” (i.e., split alpha identification) and moreover postulate that “*the localization of generators depends on the choice of the reference electrode.*” These conclusions are in stark contradiction to what is an established knowledge in both signal analysis and electrophysiology fields.

In what follows we will explain errors due to inappropriate use of spectral analysis methods and concentrate on improper interpretation of results since identifying these errors might be not so easy for readers who are not sufficiently experienced in EEG signal analysis. For example, why the “*double peaks spectrum*” was obtained for *“a window size of 2 s”* rather then for 4 s or 8 s window.

Explanation of errors is necessary for two reasons, one is an education purpose and the second one is to avoid further propagation of errors and wrong conclusions regarding “*split alpha*” in consecutive publications on the topic of EEG analysis.

## Materials and Methods

### Shortcomings of FFT Application to EEG Signal Spectral Analysis

Fast Fourier Transform (FFT) analysis (Cooley and Tukey, 1965), even though it is a commonly used method, when applied to EEG spectral analysis requires a deeper knowledge of shortcomings and properties of this method and experience, as well. The same is true for any other form of EEG analysis.

From the signal analysis point of view spontaneous EEG activity is classified as non-deterministic, stochastic process recorded as a low-voltage band limited (pink) noise and non-stationary one. The characteristic of EEG signal is influenced by various simultaneous psychophysiological processes and physical factors. Therefore, interpretation of frequency spectrum of spontaneous EEG activity should be made very carefully.

Fourier transform is an exact method only when applied to an infinite time or, at least to very long epochs in comparison to the period analyzed, and stationary signals. Otherwise, spectral analysis provides only an estimation of power spectrum. Spectral analysis is a powerful analytical tool with good statistical properties as long as certain assumptions about the nature of the data, especially normal distribution of the amplitudes and stationarity are not grossly violated. It is important to note this restriction since EEG data are rarely a Gaussian noise. Rather EEG data in many cases may look like a random noise due to EEG signal nature. This means that one must carefully examine both EEG recording as well as the resulting spectra as the error estimates of obtained spectra are difficult to obtain without the knowledge of actual frequency content. Moreover, the assumption of stationarity is valid only for relatively short epochs of EEG data. Spectrum stability and statistical significance decrease with the decrease of duration of EEG epoch under analysis.

In practice, the spectral analysis is applied to epochs (data segments) of finite and shortest possible duration (T). With short epochs one can try to minimize the effect of the EEG signal being non-stationary. However, this leads to two different sources of difficulties: spectral resolution limitation: the frequency resolution of the obtained spectrum is limited to 1/T and truncation error. In such a case the resultant spectrum is a convolution of the spectra of the epoch of EEG signal and of the time window function (Blackman-Harris, Triangle, Parzen or Hann windows, etc.). In consequence, each frequency peak is broadened into a band of 2/T width and has a series of lateral lobes, and due to truncation error harmonic frequencies may appear in the spectrum that result in spectral aliasing. Procedures of overlapping of analyzed signal epochs or spectrum smoothing might reduce spectrum alterations and increase statistical reliability of results (Kellaway and Petersen, 1973; Oppenheim and Schafer, 1989).

However, despite of objections from theory of time series analysis, spectral analysis of non-stationary EEG epochs may provide useful information and is widely used. The spectral analysis of EEG signal requires rational choice of parameters mentioned above and a large experience in the interpretation of EEG signals. Otherwise, it is easy to obtain wrong results and misinterpret the spectral content of the EEG signal. This is why automatic methods available in standard packages of numerical procedures can not be “blindly” used for calculations and making automatic inferences about the underlying brain activity.

In what follows, as example of how an incorrect use of FFT for EEG spectral analysis can result in spurious peaks which were further misinterpreted as “*split alpha*” phenomenon (see Olejarczyk et al., 2017), is presented.

## Analysis of the Factors Leading to the Emergence of Double Alpha Feature in FFT Power Spectrum

In this section we perform analysis of examples of EEG data with double alpha spectra and analyze the signal and its spectra calculated for various lengths of the data segment used. Followed by an application of model studies that enable to understand the origin of the double alpha feature.

### Analysis of Double Alpha Spectra Segments of EEG Signals

**FIGURE 1:**
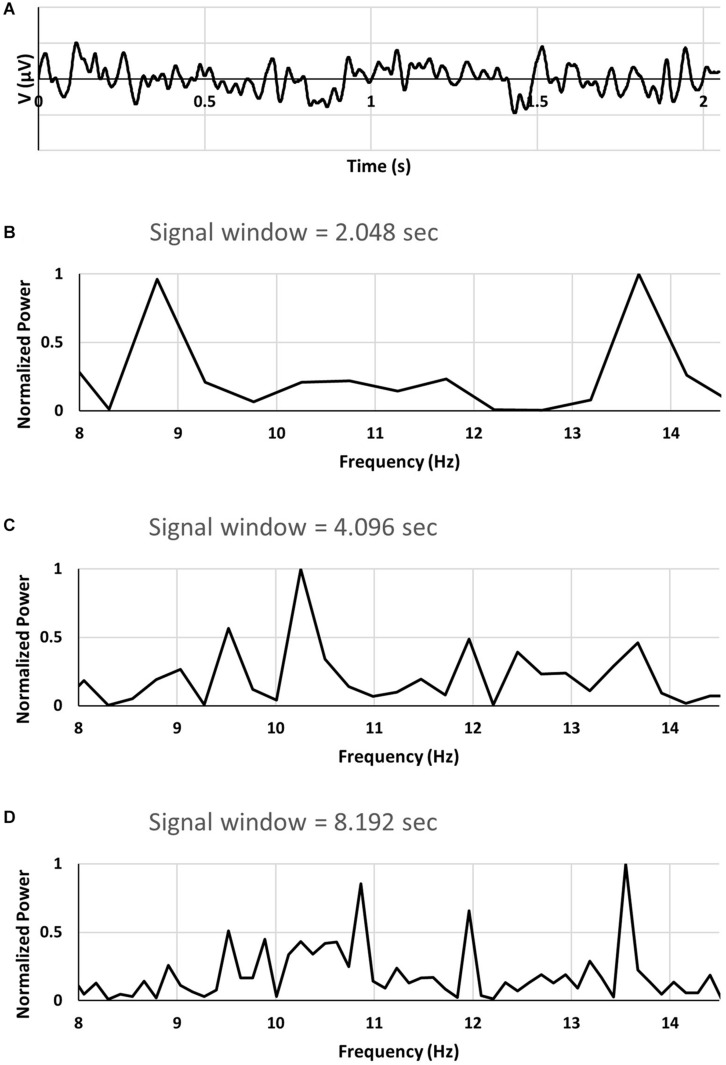
**(A)** segment of EEG signal. **(B–D)** FFT power spectra of EEG signal using signal windows of 2.048, 4.096, and 8.192 s. See text for a description.

We have analyzed an EEG recording searching for 2.048 s long segments of the signal which exhibit so called double alpha spectrum, similar to that demonstrated in Figure 1B in Olejarczyk et al. (2017). An example of EEG signal of duration 2.048 s (sampled with frequency of 1000 Hz) is shown in Figure 1A. A FFT power spectrum of the 2.048 s long EEG signal epoch shown in Figure 1A is displayed in Figure 1B. Shown in Figure 1B is a portion of the spectrum corresponding to the alpha band. As can be seen from Figure 1B the power spectrum exhibits two large peaks located at approximately 8.8 Hz and 13.7 Hz. Double alpha type spectra are found for short signal windows, typically for 2 s windows. For longer signal windows (Figures 1C,D) the spectra do not exhibit double alpha.

The spectrum for the 4.096 s signal window was calculated for the same recording as that used to find the 2.048 s segment exhibiting double alpha spectrum. For signal window of 4.096 s two consecutive 2.048 segments were used with the second segment containing the original 2.048 data.

For the 8.192 s signal window two 2.048 s segments preceding the segment with double alpha spectrum, and a single 2.048 s segment trailing were used.

From the 8.192 s signal window it is seen in Figure 1D that the spectrum contains multiple peaks as well as significant power in a wide band of frequencies. However, no significant power can be found corresponding to the two prominent peaks in the spectrum from the 2.048 signal window.

From our analyses of EEG data for other cases of double alpha spectra found it follows that the double alpha spectra appear for short signal window when the spectrum for the 8.192 s signal window shows multiple frequencies and frequency bands.

In what follows we will explore how the presence of frequency band in the EEG signal may be related to double alpha occurrence when short segments of the EEG are used for FFT analysis.

### Power Spectrum for a Signal With a Frequency Band

In order to determine what effect on power spectrum does the presence of a frequency band have the following approach may be used: instead of calculating power spectrum for a single frequency sinusoidal signal one may calculate power spectrum of a sinusoidal signal integrated over a range of frequencies.

Let *S* denote the signal obtained by integrating sinusoidal signals s(f) over a range of frequencies, from f_*a*_ to *f*_*b*_. The range of frequencies considered f_*a*_ ≤ f ≤ f_*b*_ may be considered as corresponding to the frequency band contained in the segment of the EEG signal (and visible in the spectrum in Figure 1D).

With these definitions the form for S is as follows:

(1) $$S\left(t\right)=\int_{f_{a}}^{f_{b}}s\left(f,t\right)df$$

where the sinusoid signal is given, using complex notation, by:

(2) $$s\left(f,t\right)=\sin\left(2\pi ft\right)=\frac{e^{i\omega t}-e^{-i\omega t}}{2i}$$

where the angular frequency ω = 2π*f*.

The Fourier transform of the *S*(*t*) corresponding to angular frequency ω_0_ is calculated for a finite segment of the signal of length *T* as:

(3) $$F\left(\omega_{0}\right)=\int_{0}^{T}S\left(t\right)e^{-i\omega_{0}t}dt$$

We are thus calculating Fourier transform of a windowed signal, just as is the case in case of EEG FFT analysis.

The formula for the Fourier transform may be rewritten into the following form enabling further analysis:

(4) $$\begin{array}{c} F\left(\omega_{0}\right)=\int_{0}^{T}\left(\int_{f_{a}}^{f_{b}}\frac{e^{i\left(\omega -\omega_{0}\right)t}-e^{-i\left(\omega +\omega_{0}\right)t}}{2i}df\right)dt=\\ \int_{f_{a}}^{f_{b}}\left(\int_{0}^{T}\frac{e^{i\left(\omega -\omega_{0}\right)t}-e^{-i\left(\omega +\omega_{0}\right)t}}{2i}dt\right)df\\ =\int_{f_{a}}^{f_{b}}G\left(\omega ,\omega_{0},T\right)df \end{array}$$

where $G\left(\omega ,\omega_{0},T\right)=\int_{0}^{T}\frac{e^{i\left(\omega -\omega_{0}\right)t}-e^{-i\left(\omega +\omega_{0}\right)t}}{2i}dt$ is the Fourier transform of a windowed sinusoid signal with angular frequency ω. Performing the integration the *G*(ω, ω_0_, *T*) is given by:

(5) $$\begin{array}{c} G\left(\omega ,\omega_{0},T\right)=\frac{1-e^{-i\left(\omega_{0}+\omega \right)T}}{2\left(\omega_{0}+\omega \right)}-\frac{1-e^{-i\left(\omega_{0}-\omega \right)T}}{2\left(\omega_{0}-\omega \right)}=\\ i\left(\frac{\sin\left(\left(\omega_{0}+\omega \right)T\right)}{2\left(\omega_{0}+\omega \right)}-\frac{\sin\left(\left(\omega_{0}-\omega \right)T\right)}{2\left(\omega_{0}-\omega \right)}\right)+\\ \frac{\cos\left(\left(\omega_{0}-\omega \right)T\right)}{2\left(\omega_{0}-\omega \right)}-\frac{\cos\left(\left(\omega_{0}+\omega \right)T\right)}{2\left(\omega_{0}+\omega \right)}+\\ \frac{1}{2\left(\omega_{0}+\omega \right)}-\frac{1}{2\left(\omega_{0}-\omega \right)} \end{array}$$

In order to calculate the Fourier transform corresponding to a band of frequencies the expanded formula for G is used giving for *F*(ω_0_) the following equation:

(6) $$\begin{array}{c} F\left(\omega_{0}\right)=\frac{i}{2}\left(\text{Si}\left(T\left(\omega_{0}-\omega \right)\right)+\text{Si}\left(T\left(\omega_{0}+\omega \right)\right)\right)-\\ \frac{1}{2}\left(\text{Ci}\left(T\left(\omega_{0}-\omega \right)\right)+\text{Ci}\left(T\left(\omega_{0}+\omega \right)\right)\right)+\\ {\frac{1}{2}\left(\log\left(\omega -\omega_{0}\right)+\log\left(\omega +\omega_{0}\right)\right)|}_{\omega =2\pi f_{a}}^{\omega =2\pi f_{b}} \end{array}$$

Here Ci and Si are cosine integral and sine integral, respectively. They result from the integration of expressions like cos(*x*)/*x* or sin(*x*)/*x*, and, because the integral defining F is a definite one, the *F* is calculated as the difference between the value for the integral at the upper limit and the value at the lower limit of ω. The resulting equation for *F* is quite long, however, it can be easily calculated using mathematical libraries for the calculation of sine and cosine integrals.

The resulting power spectrum *P* is calculated as:

(7) $$P\left(\omega_{0}\right)={\left|F\left(\omega_{0}\right)\right|}^{2}$$

The following graph shows power spectrum of a Fourier transform of a signal containing a band of frequencies. The range of frequencies is: *f*_*a*_ ≤ *f* ≤ *f*_*b*_, with *f*_*a*_ = 8Hz, *f*_*b*_ = 13Hz, a signal window of length *T* = 2sec.

**FIGURE 2:**
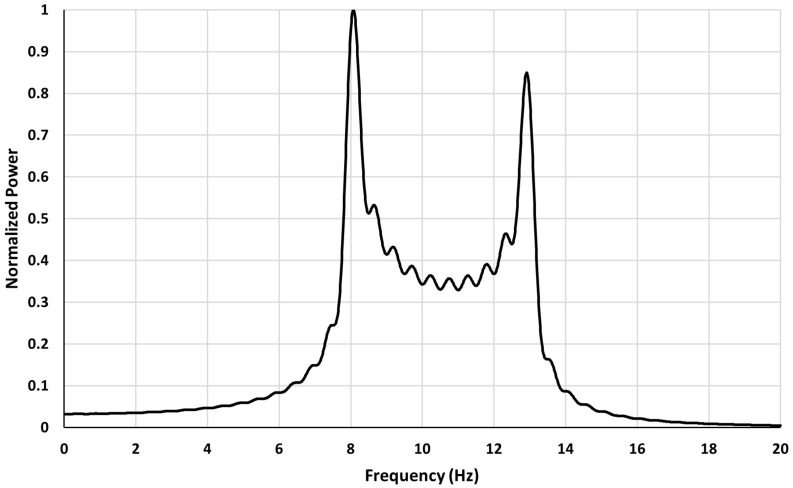
Power spectrum of F for a signal with a frequency band in the range 8–13 Hz and a signal window of 2 s.

As can be seen from Figure 2 for a signal containing a band of frequencies the power spectrum when calculated from a finite segment of signal exhibits double peak feature. The peaks occur at the edges of the assumed frequency band (8–13 Hz). The wobbles seen on the graph result from the oscillations present in Ci and Si.

Therefore, a power spectrum of a signal which contains a band of frequencies results in the emergence of peaks in the spectrum at the edges of the frequency band. These peaks have no relation to any frequencies of the signal and are an effect of spectrum leakage.

### Effect of Windowing Data Before FFT Calculation

The signal analyzed in Section “Power Spectrum for a Signal With a Frequency Band” was not windowed and in order to improve the performance of Fourier transform the so called windows are frequently used. The window function used is usually symmetric around the middle of signal’s segment and falls to zero at the edges of the segment.

In order to find out how windowing affects the shape of the spectrum a simulation was performed in which a sum of 22 sinusoids was calculated with the frequencies of the sinusoids spaced evenly in a range 8–13 Hz. The signal was sampled with a frequency of f_*s*_ = 100 Hz, and the segment length *T* was 2.56 s (i.e., 256 samples were used to calculate FFT).

The analyzed model signal used may be described using following formula:

(8) $$y\left(t_{j}\right)=\sum_{i=1}^{n}y_{i}\left(t_{j}\right)=\sum_{i=1}^{n}\text{sin}\left(2\pi f_{i}t_{j}\right)$$

where *f*_*i*_ = *f*_*a*_ + *i*⋅δ*f*, with δ*f* being the frequency step (in Hz), *f*_*a*_ the lower frequency of the range, the *f*_*b*_ the upper frequency range and *i* = 1..*n*, such that *f*_*a*_ ≤ *f*_*i*_ ≤ *f*_*b*_, and where n can be selected (here *n = 22*). The *t*_*j*_ = *j*/*f*_*s*_, with *f*_*s*_ being the sampling frequency, and *j* = 0..*N*−1.

**FIGURE 3:**
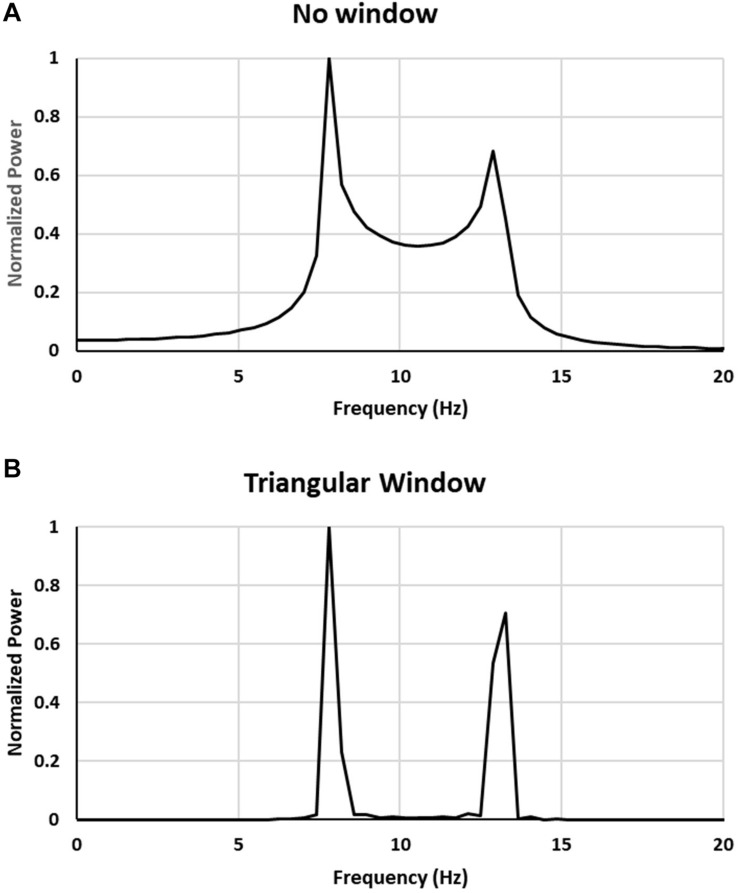
Comparison of power spectra for a sum of 22 sine waves with frequencies equally spaced in 8–13 Hz range: **(A)** no window, **(B)** triangular window applied to signal.

Before submitting the signal to FFT analysis it was passed through either a rectangular window (this did not change the signal) or a Blackman-Harris, Triangle, Parzen or Hann windows. The results are presented in Figure 3 for rectangular (no window) and Triangle windows.

As can be seen from Figure 3A also for a signal composed of a finite number of frequency components (22 in this case) the resulting power spectrum closely resembles the one obtained for a modeled signal with a continuous range of frequencies (frequency band) shown in Figure 2 thus showing that spurious peaks in the spectrum appear also for a signal with a finite number of frequencies.

By filtering the signal using triangular window before applying FFT only leads to amplification of the magnitude of the peaks appearing at the edges of the frequency interval. The same effect occurs for other windows that we have tried. Thus, by windowing the signal only the amplification of spurious peaks occurs.

**FIGURE 4:**
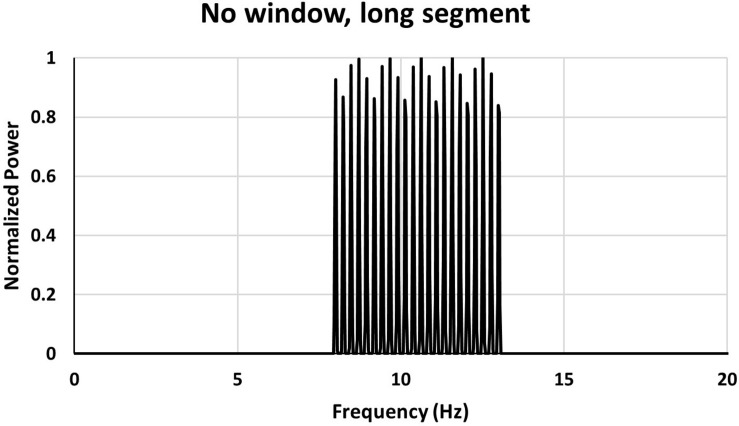
Power spectrum for a long data segment (40.96 s). The same signal as used in Figure 3.

If on the other hand one uses a long segment the power spectrum of the same signal as used in Figure 3 looks like in Figure 4.

With a long signal segment (40.96 s in the case shown in Figure 4) individual peaks appear and one can see from the spectrum that the signal is composed of several frequencies and there are no spurious peaks at the edges of the frequency band.

It may also be interesting to examine what should be the number of frequencies present for the double alpha to appear in the spectrum. It turns out that for a f_*s*_ = 100 Hz, signal length *T* = 2.56 s case it is sufficient to sum 13 sinusoids with frequencies equally spaced in the 8–13 Hz range to obtain double alpha spectrum. And by a few frequencies less if a Triangle window (or any other of the considered types) is used.

### Effect of a Definitive Two Frequencies in the Signal

Up till now we have analyzed the power spectra obtained from a continuous or discrete set of frequencies with same amplitude sine waves. It is also worthwhile to examine the power spectrum obtained for a short duration segment when there are two dominant frequencies as this is what was expected when the double alpha spectra were discovered. Since in order for the double peaks to appear several (>10) frequencies are necessary we have computed a simulated signal that was composed of two sets of frequencies: *f*_1_ = 10.48Hz and *f*_2_ = 11Hz. With a sampling frequency *f*_*s*_ = 100Hz and segment length of: 2.048, 4.096 or 8.192 s. Even with the shortest segment length the frequency resolution is sufficient to resolve the two main peaks. Each of the groups of frequencies was accompanied by ±5 accompanying frequencies, spaced 0.41 Hz apart so that in the two groups the frequencies were: f_1i_ =10.48+0.41i, and *f*_2*i*_ = 11 + 0.41*i*, with −5≤*i*≤5. Altogether then there were 21 frequencies present covering a range of 5.89–12.53 Hz for the first group, and 8.59–13.05 Hz for the second group. The amplitude of the sinusoidal components was not constant it decreased from 1 for i=0 to 0.2 for *i* = ±5. As can be seen the frequency ranges of the two groups were overlapping and were sufficiently close together to result in a double alpha peaks.

**FIGURE 5:**
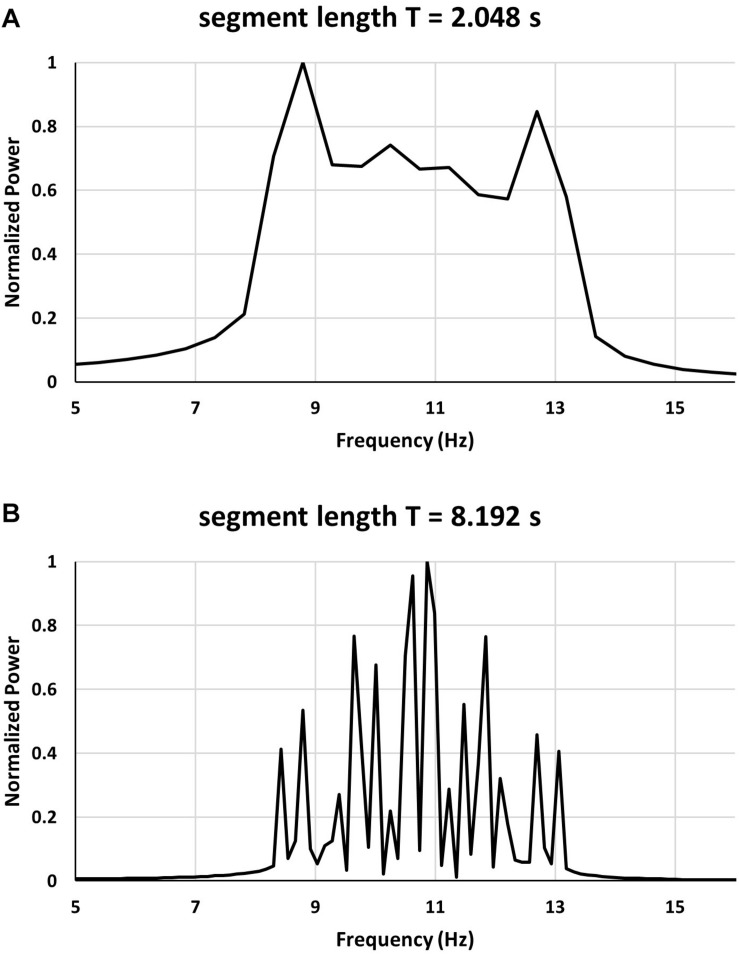
Comparison of two power spectrum of the same multifrequency signal with two dominant frequencies for short and long segment length.

The power spectrum obtained for this case is seen in Figure 5. As can be seen from Figure 5 in case of the short segment length (2.048 s) there appear the characteristic double peaks – one located at around 8.86 Hz and the second at around 12.74 Hz. In the longer segment (length equal to 8.192 s) the large number of peaks with the dominant peaks occurring at around 10–11 Hz is seen. For the 2.048 s segment the two spurious peaks correspond to the inner range of overlapping frequencies (8.59–12.53 Hz) confirming that the double peaks are due to the spectrum leakage and frequency overlap and do not correspond to the actual frequencies present in the signal.

### Some Practical Indications

When in an epoch of EEG signal several closely spaced frequencies appear so that a frequency band emerges this forms the conditions for the formation of characteristic double peaks spectrum.

The double peaks appear as a result of interference from the spectra from individual frequencies which are widened by the finite epoch duration. And they appear at the boundaries of the frequency band because of Gibbs phenomenon (Gibbs, 1898; Hewitt and Hewitt, 1979).

The emergence of double peaks depends sensitively on quantities like relative phases of the frequency components, amplitudes of these components and other parameters. This means not for every multifrequency signal will the double peaks appear. However, when they appear close to the ends of the band it is a strong indication that these are not due to real frequencies.

A single frequency for an epoch of duration T seconds contributes to power spectrum with a peak of width of about 1/*T* Hz, although the actual width depends on the used window.

Two closely spaced peaks will result in power spectrum that will be the sum of their spectra, and if they are spaced closer than about half-width of their peaks their combined spectrum will be affected by interference. Hence, a simple criterium for the appearance of interference is that the frequencies are spaced closer than: Δ*f*_*i*_ = 1/*T* Hz.

The peaks around 8 Hz and 13 Hz seen in Figure 2 are due primarily to the sine integral (Si) or cosine integral (Ci) terms present in equation 6 for *F*(ω_0_) in Section “Power Spectrum for a Signal With a Frequency Band.” The primary fluctuations of Si or Ci have a half width of about 6. If we assume that the primary fluctuations of these terms are responsible for the formation of the side peaks located at the edges of the band then the width of the side peak (Δ*f*_*w*_) may be related to epoch duration as: Δ*wT* = 2πΔ*f*_*w*_ ≈ 6.

The resulting frequency width (Δ*f*_*w*_) is: $\Delta f_{W}=\Delta w/2\pi \approx \frac{6}{2\pi T}\approx 0.95/T$ Hz. The peak is thus comparable in width to the sampling frequency (1/*T*), although somewhat narrower.

For the two side peaks not to merge the minimum band width (Δ*f*_*B*_ Hz) must be larger than twice the side peak width: Δ*f*_*B*_ = 2Δ*f*_*w*_≈ 1.9/T Hz.

If the frequencies are spaced closer than the minimum band width Δ*f*_*B*_ then the resulting spectrum will contain a single wide peak that is a result of merging of all peaks in the band into a single wide peak and no double peak spectrum will appear.

On the other hand, if there are sufficiently many frequencies and they are spread over a band of width larger than Δ*f*_*B*_ this forms conditions for the emergence of double peaks. The number of frequencies (N) that need to occur in a band Δ*f*_*B*_ for the split alpha to emerge in the spectrum may be estimated from: N = $\frac{\Delta f_{B}}{\Delta f_{i}}\approx$ 2.

Please note how narrow is the minimum band width. In EEG the split-alpha bands have much larger width, implying presence of much larger number of frequencies than the minimal 2.

If there are more frequencies they may be spread over a wider band provided they remain spaced no more than Δ*f*_*i*_ Hz apart.

If there are fewer frequencies than N in a band of width Δ*f*_*B*_ or they are spread over broader frequency range than Δ*f*_*B*_ the split-alpha effect will not appear.

On the other hand if one observes double peaks in an EEG segment and the side peaks are separated by *B* Hz and B < Δ*f*_*B*_ then one can suppose that the peaks are due to two frequencies and are not a manifestation of a multi-frequency EEG event.

From the above it follows that in order to check if an observed double peaks is due to a multiple frequency EEG or due to two frequencies it is necessary to compute power spectrum with a four times longer epoch.

If we take a four times longer EEG segment then the minimum frequency band for such longer segment is four times narrower. Since taking four times longer segment does not increase the number of frequencies present (assuming EEG signal does not change significantly) then it follows that in a four times narrower band there will be approximately four times less frequencies present and hence there will be too few frequencies to produce “split-alpha” like spectrum.

Of course, it may so happen that the EEG will contain large number of frequencies and taking a four times longer epoch will not be sufficient, but in practice it is found that comparison of power spectra from the original and two times longer segment already provides clues to the nature of the observed double peaks event, and usually four times longer segment is sufficient to verify whether double peaks are due to multiple frequencies or two.

As an example, we may consider a segment of EEG of length *T* = 2.56 s (as in Figure 2). For this case, the Δ*f*_*B*_ = 1.6/*T* = 0.63 Hz and spectrum frequency resolution is equal: 1/*T* = 0.39 Hz. If double peaks appear in a separation substantially smaller than Δ*f*_*B*_ then one may expect them to be due to two frequencies. However, such situation is not very likely because the band width is really small.

If the observed double peaks width is larger than Δ*f*_*B*_ then one may suppose it is an artifact that appears in Fourier spectra due to Gibbs phenomenon rather than so called “split alpha phenomenon.”

When one applies time domain windowing to improve time resolution the frequency resolution of the spectrum decreases, that is the spectral peaks become wider. In the present case this has such implication that fewer frequencies in a band are necessary for the double peaks effect to occur (this effect is seen in Figure 3B). Hence, using windows of the type considered here does not help in determining whether the double peaks are real.

However, as may be seen from Figure 2 or Figure 3A in the frequency range between the two side peaks there is a plateau due to multiple frequencies. Thus, one of the signs indicating that we are dealing with a spurious double peaks is when they occur superposed over a continuous power background and are located near the ends of that continuous power band.

## Discussion

In Section “Analysis of the Factors Leading to the Emergence of Double Alpha Feature in FFT Power Spectrum” it has been shown that when the analyzed EEG signal contains multiple frequencies there appear spurious peaks at the edges of the frequency band. This occurs when the FFT is computed for a short data segment (like 2 s). For short data segments both the frequency resolution of the spectrum is low (it is inversely proportional to an epoch of signal length in seconds) and there appears distortions in the calculated spectrum at the end of frequency band that are a result of spectrum leakage. These two factors work together to obliterate the real nature of the spectrum. In order to obtain spectrum free of this distortion care has to be taken that the FFT provides real information (by for example examining longer periods of signal or analyzing EEG signal when there are no pronounced frequency bands present etc.).

The reported in the literature by Olejarczyk et al. (2017) beneficial effect of using short signal segments (of 2 s) as this enables to observe the so called “*split alpha*” does not in fact enable any deeper insight into the nature of the examined EEG recording, it merely is a result of power leakage due to low resolution and properties of the Fourier transform. It thus has no diagnostic meaning to analyze double alpha other as a precaution against using too short data segment for the analysis or as an indication that the analyzed EEG signal contains multiple closely spaced frequencies (or a frequency band).

The misinterpretation by the authors (Olejarczyk et al., 2017) of EEG signal analysis results shown in Figure 1B in Olejarczyk et al. (2017) has led them to wrong conclusions. Authors tried to interpret these two spurious peaks in spectrum, similar to those shown in Figure 1A above, as signals from two “distinct” generators and then proceeded to localize these “new generators” in the brain using DTF method. However, the so called “*split alpha*” is a result of serious methodological errors, as it was shown in Section “Analysis of the Factors Leading to the Emergence of Double Alpha Feature in FFT Power Spectrum.”

In conclusions the authors stated that “*the split alpha spectrum can be generated by two or more independent and interconnected alpha wave generators located in different regions of the cerebral cortex, but not necessarily in the occipital cortex*” and “*a window size of 2 s was found to be optimal for this purpose”* (i.e., split alpha identification) and moreover postulate that “*the localization of generators depends on the choice of the reference electrode.*”

Unfortunately, these conclusions might sound attractive and therefore might be repeated and propagated by readers which are not critical enough (Dauwan et al., 2018; Viereck, 2018; Korovesis et al., 2019; Ustinin et al., 2020).

To illustrate above some citations from the paper Olejarczyk et al. (2017): “….*Both cases discussed above illustrate the existence of generators localized in posterior areas, particularly at derivations O1 and O2. Alpha rhythms can be also generated in other parts of the brain. The coexistence of several generators was demonstrated in the third patient, a 54-year-old woman [see Figure 4 in Olejarczyk et al. (2017)]. The localization of generators depends on the choice of the reference electrode*.” and more: “*In addition, other generators were identified when the reference was placed on the neck, with one at P8 (with maximum strength at 9–10 Hz) and another at O2 (with maximum strength at 11–12 Hz). These DTF results changed dramatically when the signal was rereferenced to the linked earlobes [cf. Figure 6B in Olejarczyk et al. (2017)]. A second generator was identified at electrode Pz, which dominated for frequency of 10 Hz*.”

This illustrates that the attempts to assign physiological meaning to the events of “*split alpha*” made without the examination of the sources of such double peaks emergence can lead to incorrect conclusions.

We do not comment on any statements provided in the cited paper regarding neurophysiological interpretations of results. Also, the issue of property of application of DTF method for “*split alpha*” generators localization as well as other methodological bugs are out of the scope of this paper. False conclusions such as, i.e., “*influence of reference electrode on generator localization*” are more than obvious since understanding the role of reference electrode is a fundamental to anyone attempting electrophysiological measurements in biomedical engineering. In any case the reference electrode can not influence localization of generator in the brain.

### General Remarks

The above analysis and conclusions authorize us to some general remarks on EEG analysis. Visual inspection of EEG recordings in the time domain is an empirical science and requires a considerable amount of clinical and neurophysiological knowledge. Any methods of automatic analysis can serve as a support which might be helpful in EEG recording evaluation but only in case when all shortcomings and properties of this method are very well understood. FFT analysis is a good example and from the very beginning an assumption that EEG is a phenomenon to which Fourier transform can usefully be applied was questionable and spectral analysis of EEG has to be applied and interpreted very carefully. It is not possible to assume that the spectra show “true” EEG features without a deeper analysis of the EEG signal and the conditions in which the spectra were obtained. In a sense the EEG signal is not a good candidate for a fully automatic spectral analysis as it is easy to obtain completely incorrect results (as we have shown above).

The paper on “*split alpha*” is not an exception in the area of incorrect use of automatic EEG signal analysis, however, it is strongly worth to clarify these errors because definition of “new phenomena” such as “*split alpha*” and identification by authors of “new generators” in the brain the localization of which may change due to a change of electrode selected for reference does not progress the understanding of the nature of EEG and may easily lead others to pursue non-existing phenomena.

## Conclusion

We have shown the rationale behind a “new phenomenon” of so called “*split alpha*” described in Olejarczyk et al. (2017). From our analyses of EEG data for cases of double alpha spectra it follows that the double alpha spectra can appear, as spurious peaks, for short signal window when the EEG signal under study shows multiple frequencies and frequency bands.

Therefore, a power spectrum of a signal which contains a band of frequencies results in the emergence of peaks in the spectrum at the edges of the frequency band. These peaks have no relation to any frequencies of the signal and are an effect of spectrum leakage. Any attempt to use it to localize generators of alpha activity in brain is pointless.

Hence, the emergence of peaks at the ends of a frequency band is a spurious effect caused by the leakage of power in the spectrum obtained from a discrete, short epoch EEG recording. It is important that there should be multiple frequencies (or a continuous frequency band) present in the recorded segment as this is the factor that leads to the emergence of double peaks located off-center, close to the ends of the band. The same effect of double peaks emergence is not a feature of the alpha frequency band, it may appear for any other band too, provided that there is concentration of frequencies sufficiently close together (a band of frequencies).

At the end one might point the following highlights:

1. The double alpha EEG spectra called as “*split alpha*” can appear in the form of two spurious peaks with no relation to any frequencies of the signal and is an effect of spectrum leakage.
2. Double peaks like spectrum in any frequency band may be a result of FFT method properties when applied to short time signal of multiple frequencies, i.e., Gibbs phenomenon.
3. The automatic methods available in standard packages of numerical procedures, even dedicated for EEG analysis, can not be “blindly” used for calculations and making automatic inferences about the underlying brain activity.

## Data Availability Statement

The datasets presented in this article are not readily available because the manuscript contains theoretical calculations and analysis on simulated data. Requests to access the datasets should be directed to EZ, ewa.zalewska@ibib.waw.pl.

## Author Contributions

The author confirms being the sole contributor of this work and has approved it for publication.

## Conflict of Interest

The author declares that the research was conducted in the absence of any commercial or financial relationships that could be construed as a potential conflict of interest.
